# Glutamate May Be an Efferent Transmitter That Elicits Inhibition in Mouse Taste Buds

**DOI:** 10.1371/journal.pone.0030662

**Published:** 2012-01-26

**Authors:** Yijen A. Huang, Jeff Grant, Stephen Roper

**Affiliations:** 1 Department of Physiology and Biophysics, University of Miami School of Medicine, Miami, Florida, United States of America; 2 Program in Neuroscience, University of Miami School of Medicine, Miami, Florida United States of America; Duke University, United States of America

## Abstract

Recent studies suggest that l-glutamate may be an efferent transmitter released from axons innervating taste buds. In this report, we determined the types of ionotropic synaptic glutamate receptors present on taste cells and that underlie this postulated efferent transmission. We also studied what effect glutamate exerts on taste bud function. We isolated mouse taste buds and taste cells, conducted functional imaging using Fura 2, and used cellular biosensors to monitor taste-evoked transmitter release. The findings show that a large fraction of Presynaptic (Type III) taste bud cells (∼50%) respond to 100 µM glutamate, NMDA, or kainic acid (KA) with an increase in intracellular Ca^2+^. In contrast, Receptor (Type II) taste cells rarely (4%) responded to 100 µM glutamate. At this concentration and with these compounds, these agonists activate glutamatergic synaptic receptors, not glutamate taste (umami) receptors. Moreover, applying glutamate, NMDA, or KA caused taste buds to secrete 5-HT, a Presynaptic taste cell transmitter, but not ATP, a Receptor cell transmitter. Indeed, glutamate-evoked 5-HT release inhibited taste-evoked ATP secretion. The findings are consistent with a role for glutamate in taste buds as an inhibitory efferent transmitter that acts via ionotropic synaptic glutamate receptors.

## Introduction

During taste stimulation, important synaptic interactions (paracrine and autocrine) take place in the taste bud between the different cell types. Recent studies have implicated several neurotransmitters and signaling molecules in these interactions, including serotonin, ATP, norepinephrine, GABA, acetylcholine, cholecystokinin, and neuropeptide Y [1]–[14]. Receptor (Type II) cells, which express G-protein-coupled receptors (GPCRs) for bitter, sweet, and umami [15]–[18], secrete ATP in response to taste stimulation or depolarization [6], [12]. Indeed, ATP is believed to be a key excitatory transmitter between taste receptor cells and gustatory sensory afferent fibers [4], [19]. Presynaptic taste cells, which respond to sour (acid) taste stimuli, secrete serotonin, norepinephrine, and GABA [5], [7], [8], [11], [20] during taste stimulation.

Physiological and immunochemical studies indicate glutamatergic signaling occurs in taste buds as well. Rat lingual epithelium containing taste buds expresses mRNA for NMDA- and kainate-type glutamate receptors [21]. Our laboratory and others have demonstrated that NMDA [22]–[24] and kainic acid [24] excite taste cells, indicating the presence of NMDA- and kainate-type glutamate receptors in taste buds. It was recently reported that mouse taste buds express subunits for kainate-type glutamate receptors and that glutamate at 1 to 100 mM stimulated Presynaptic (Type III) taste cells, with the conclusion that this excitatory amino acid was an efferent transmitter onto those cells [25]. Consistent with that interpretation, vesicular glutamate transporters are found in fibers innervating mouse taste papillae, suggesting that glutamate is likely released from these fibers onto taste buds [25]. Collectively, these studies point to a role for glutamate as a neurotransmitter in the peripheral taste pathway, although the detailed actions of glutamate in the taste bud at concentrations that unambiguously discriminate synaptic *versus* taste receptors remains to be tested.

In this study, we used Ca^2+^ imaging to determine which specific type(s) of taste cells express functional synaptic glutamate receptors and how excitation of these synaptic receptors affects taste responses. We show that many Presynaptic cells respond to the NMDA-receptor agonist NMDA as well as the AMPA/kainite receptor agonist, kainic acid. Furthermore, activation of these ionotropic glutamate receptors stimulates taste buds to release serotonin and inhibit taste-evoked ATP secretion, demonstrating that synaptic glutamate can modify the signal output from taste buds.

## Materials and Methods

### Animals and Ethical approval

Mice were killed following National Institutes of Health guidelines and all experimental procedures were approved by the University of Miami Animal Care and Use Committee. Adult C57BL/6J mice, transgenic mice expressing enhanced green fluorescent protein (GFP) under control of the PLCβ2 promoter (PLCβ2–GFP) [26], or transgenic mice expressing GFP under the control of the GAD67 promoter (GAD67-GFP) [27] were euthanized by exposure to 100% CO_2_ followed by cervical dislocation. This procedure minimizes distress (NIH Office of Animal Care and Use, http://oacu.od.nih.gov/ARAC/EuthCO2.pdf). Tongues were removed for further dissection.

### Isolated taste buds and taste cells

Lingual epithelium containing vallate mouse papillae was removed from the tongue by injecting an enzyme mixture (1 mg ml^−1^ collagenase A, Roche), 2.5 mg ml^−1^ dispase II (Roche), 0.25 mg ml^−1^ Elastase (Worthington), and 0.5 mg ml^−1^ DNAse I (Sigma) directly under the epithelium surrounding the papillae. Twenty minutes later the epithelium was peeled from the tongue, re-incubated for 2 min in fresh enzyme mixture, and 5 min in Ca^2+^/Mg^2+^-free Tyrode solution. Taste buds were carefully removed from the serosal surface by gentle suction into a fire-polished micropipette and transferred to a recording chamber. To obtain single taste cells, isolated taste buds were incubated for 10 min in 0.25% trypsin and then triturated 20 times with a fire-polished micropipette. An aliquot of isolated cells was transferred to the recording chamber and cells were loaded with 5 µM Fura 2 AM (Invitrogen). During the experiment, taste buds and taste cells were continuously perfused with Tyrode solution (in mM: 140 NaCl, 5 KCl, 2 CaCl_2_, 1 MgCl_2_, 10 HEPES, 10 glucose, 10 sodium pyruvate, 5 NaHCO_3_, pH 7.2–7.4, 310–320 mOsm/l). For experiments to detect serotonin (5-HT) release, whole taste buds or isolated taste cells were pre-incubated with 5-hydroxy-tryptophan (500 µM) for 30 min prior to the start of the experiment to maximize 5-HT loading of taste cells, as reported in Huang et al. [5].

### Biosensor cells

CHO cells co-expressing 5-HT_2C_ receptors and purinergic P2X2/P2X3 receptors (dual biosensors) were prepared and loaded with 5 µM Fura 2 AM (Invitrogen) as described previously [6]. To test for 5-HT secretion, purinoreceptors were desensitized by incubating biosensors with 500 µM ATP for 30 min prior to experiments. Conversely, to test for ATP secretion, 5-HT receptors on the biosensors were desensitized by incubation with 1 mM 5-HT for 30 min. These procedures are described in detail in Huang et al. [6], [7]. Biosensor cells alone (i.e., in the absence of taste buds) did not respond to any of the glutamatergic compounds used in this study and their sensitivities to ATP or 5-HT were unaffected by the pharmacological agents we employed [6], [28].

### Ca^2+^ imaging

Ca^2+^ imaging was carried out as described fully in Dvoryanchikov et al. [11]. F340/F380 ratios were converted to Ca^2+^ concentration values using a Fura 2 calcium calibration buffer kit (Invitrogen, Carlsbad, California) as follows:
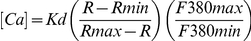
with [Ca^2+^] in nM; Kd = 224 nM [29]; R = measured ratio (F340/F380); R_min_ = ratio at zero free Ca^2+^; R_max_ = ratio at saturating Ca^2+^ (39 µM); F380_max_ is the fluorescence intensity at λ = 380 nm in zero Ca^2+^; and F380_min_ is the fluorescence intensity at λ = 380 nm in saturating Ca^2+^.

Receptor (Type II) cells were identified by their responsiveness to a sweet-bitter tastant mixture in wild-type mice or by their green fluorescence when isolated from taste buds of transgenic PLCβ2-GFP-expressing mice [26]. Presynaptic (Type III) cells were identified by Ca^2+^ influx when depolarized with 50 mM KCl in wild-type mice or by fluorescence when isolated from taste buds of transgenic GAD67-GFP mice [30].

For quantification, responses were measured as peak [Ca^2+^] minus the immediately preceding baseline (i.e., Δ[Ca^2+^] from baseline). To improve reliability and consistency of the measurements, quantification was carried out on a moving average (n = 3 points) of the raw data ([Ca^2+^]). A cell was categorized as responding to an applied agonist (e.g. glutamate) if the peak Δ[Ca^2+^] was >twice the mean baseline Ca^2+^ fluctuation.

### Stimulation

Isolated taste buds and taste cells were stimulated by bath-perfusion of KCl (50 mM, substituted equimolar for NaCl), taste mix (10 µM cycloheximide, 2 mM saccharin, 0.1 mM SC45647, 1 mM denatonium), glutamate (30–100 µM), NMDA (30–100 µM, Tocris), or kainic acid (3–100 µM, Tocris). Stimuli were bath-applied for 30 seconds followed by return to buffer perfusion for at least 3 min. This procedure produced reliable and stable stimulus-evoked responses from taste buds and isolated taste cells. To optimize conditions for NMDAR activation, Mg^2+^ was removed from the buffer (substituted equimolar with Ca^2+^) and the NMDAR co-agonist glycine (30 µM) was added.

## Results

### Glutamate activates synaptic glutamate receptors in taste cells

Previous studies have revealed the presence of both NMDA- and kainate-type glutamate receptors on taste cells [21]–[24]. Recently, Vandenbeuch et al. [25] showed that kainate-type glutamate receptors are present specifically on Presynaptic (Type III) taste cells. We began our study by replicating and refining those findings, using lower concentrations of receptor agonists (e.g., 100 µM glutamate) to avoid any confusion with glutamate taste (umami) receptors, which have a threshold >1 mM glutamate [31]–[34].

**Figure 1:**
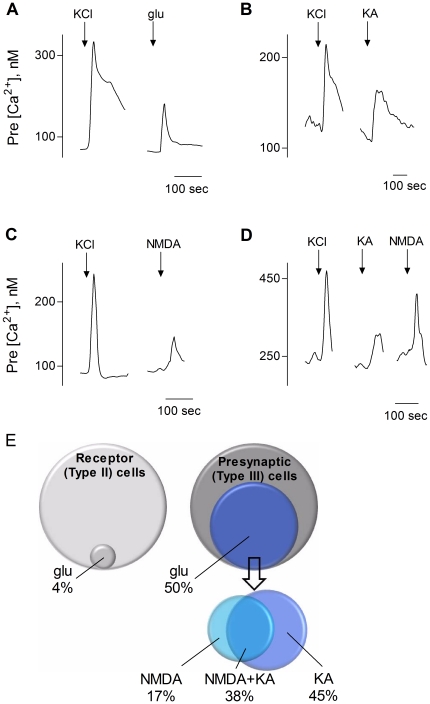
Presynaptic (Type III) taste bud cells respond to glutamate, kainic acid (KA), and NMDA. Taste cells were isolated from mouse circumvallate papillae and their responses to ionotropic glutamate receptor agonists recorded by Ca^2+^ imaging. **A**, Representative traces of an identified Presynaptic (Type III) taste cell depolarized by KCl (50 mM) (↓, KCl) followed by stimulation with glutamate (100 µM) (↓, glu) **B**, Another Presynaptic cell responded to KCl depolarization (↓, KCl) and 100 µM KA (↓, KA). **C**, A different Presynaptic cell responded to KCl (↓, KCl) and to 100 µM NMDA (↓, NMDA). **D**, In another Presynaptic cell, responses were evoked by KCl depolarization (↓, KCl), KA (↓, KA), and NMDA (↓, NMDA) alike. Note, as shown in all records in this figure, KCl stimulation typically elicited more robust responses than did glutamate, KA, or NMDA. **E**, Venn diagrams representing the relative proportions of Receptor and Presynaptic taste cells that responded to glutamate, as well as the overlap of responses of glutamate-sensitive Presynaptic cells to NMDA and/or KA.

Approximately 25% of all isolated taste cells (31/138) exhibited Ca^2+^ responses when stimulated with bath-applied glutamate (100 µM). Of the cells specifically identified as Receptor (Type II) cells, only 4% (2/50) showed Ca^2+^ responses to glutamate. In marked contrast, 50% of identified Presynaptic (Type III) cells responded to glutamate (21/42, Fig. 1A). We next conducted a series of experiments to test the effects of kainic acid (KA), an AMPA/Kainate receptor agonist, and NMDA on Presynaptic cells. We initiated these experiments using KA or NMDA stimulation alone to prevent possible desensitization or other, unknown interactions between trials. KA (100 µM) elicited Ca^2+^ responses, but in fewer Presynaptic cells than did glutamate (23%, 7/31; Fig. 1B). NMDA (100 µM), too, triggered small but reliable Ca^2+^ responses in 13% of Presynaptic cells (16/120, Fig. 1C). Lastly, in a final series of experiments to examine overlap between KA- and NMDA-sensitivity, we applied KA and NMDA in alternating sequence and with thorough rinses between trials. Of all Presynaptic cells that responded to NMDA or KA or both, 45% (13/29) responded only to KA, 17% only to NMDA (5/29), and 38% (11/29) responded to both (Fig. 1D). Figure 1E summarizes these experiments and shows the relative proportions of taste cells that respond to glutamate, KA, and NMDA.

### Glutamate, NMDA, and kainate induce transmitter release from taste buds

**Figure 2:**
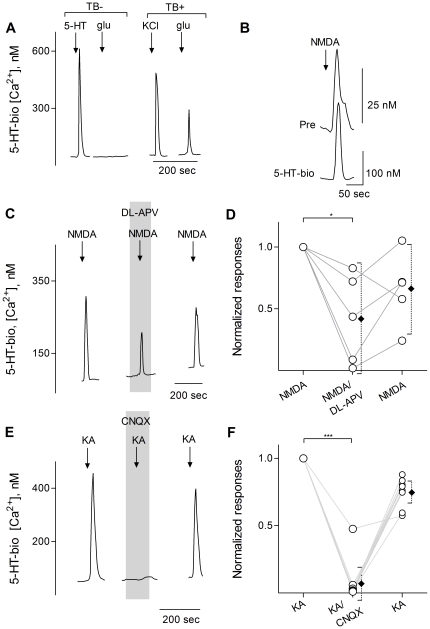
Glutamate, NMDA, and kainic acid induce serotonin release from isolated taste buds and cells. Serotonin (5-HT) biosensors were positioned against circumvallate taste buds to measure stimulus-evoked transmitter release. **A**, Traces show biosensor responses. When the biosensor was not near a taste bud (TB-), the biosensor responded only to 3 nM 5-HT (↓, 5-HT) but not to 100 µM glutamate (↓, glu) or KCl depolarization (not shown), verifying that the biosensor did not respond to stimuli that activate taste buds. In contrast, when the biosensor was positioned against a taste bud (TB+), KCl depolarization (↓, KCl) and glutamate alike (↓, glu) elicited biosensor responses, indicating stimulus-evoked 5-HT release. **B**, Simultaneous recordings from an isolated Presynaptic cell (top trace, Pre) and a 5-HT biosensor (bottom trace, 5-HT-bio). Stimulating the Presynaptic cell with 30 µM NMDA (↓, NMDA) triggered 5-HT secretion, as evidenced by the robust biosensor response (bottom). **C**, In another experiment, NMDA (↓, NMDA) (30 µM) triggered 5-HT release from a taste bud. The NMDA-evoked release of 5-HT was reversibly reduced by DL-APV (15 µM, present throughout shaded area). **D**, Summary of NMDA-evoked 5-HT release before, during and after the presence of DL-APV. Open circles represent normalized peak biosensor responses. Offset closed symbols show mean ± 95% Confidence Interval (95% CI). *, p<0.05, repeated measures ANOVA, N = 5). **E**, Kainic acid (↓, KA) (3 µM) also induced 5-HT release from a taste bud. KA-induced 5-HT release was reversibly inhibited by CNQX (30 nM, present throughout shaded area). **F**, Summary of experiments testing CNQX, plotted as in D. ***, p<0.001, repeated measures ANOVA, N = 9).

Taste buds, and specifically Presynaptic (Type III) cells, secrete the neurotransmitter 5-HT when stimulated with tastants [5]–[7]. Accordingly, because glutamate mainly activated Presynaptic cells, we tested whether glutamate also induced taste buds to release 5-HT. Using biosensors, we showed that depolarizing taste buds with 50 mM KCl triggers 5-HT secretion, as previously demonstrated [5]. Out of 49 taste buds that secreted 5-HT in response to KCl depolarization, 14 also released 5-HT when stimulated with 100 µM glutamate (Fig. 2A). Moreover, NMDA (30 µM) (Figs. 2B,C) or KA (3 µM) (Figs. 2E) also triggered 5-HT release. To verify that these agonists were activating their cognate receptors on taste cells, we applied the specific NMDA receptor antagonist DL-APV (15 µM), or specific AMPA/Kainate receptor antagonist CNQX (30 nM), and retested NMDA and KA. DL-APV significantly and reversibly decreased NMDA-induced 5-HT release from taste buds (Figs. 2C, D). CNQX significantly inhibited KA-induced 5-HT release (Figs. 2E, F). In separate experiments, the combination of CNQX and DL-APV reversibly and completely inhibited glutamate (100 µM)-elicited 5-HT from isolated taste buds (data not shown). Controls showed that in the absence of taste buds, 5-HT biosensors did not respond to any of the compounds used for this study except, of course, for 5-HT.

To verify that glutamate, KA, and NMDA specifically stimulated Presynaptic (Type III) cells to secrete 5-HT, consistent with the ability of the agonists to activate Ca^2+^ transients in these cells (Fig. 1), we isolated individual cells and tested them with 5-HT biosensors. Identified single Presynaptic taste cells, if responding to glutamate, KA, or NMDA, secreted 5-HT in response to stimulation with those agonists. Biosensors were able to detect 5-HT release in 2 out of 6 isolated Presynaptic cells that responded to glutamate. Similarly, biosensors detected 5-HT secretion from NMDA- (4/7) and KA- (3/8) responsive Presynaptic cells. As an example, Figure 2B illustrates NMDA-stimulated 5-HT secretion from an isolated Presynaptic cell. Parenthetically, the observed incidence of glutamate-, KA-, and NMDA-evoked 5-HT secretion is certainly a gross underestimate of the true incidence. Successful detection of transmitter secretion with this technique requires accurate positioning of biosensors against transmitter release site(s), which are, of course, not visible and only found by trial and error. Although we carefully maneuvered biosensors against isolated taste cells and tested more than one apposition, it is not possible to systematically scan an entire isolated taste cell for possible release sites, hence the underestimate.

### Glutamate-induced serotonin release blocks ATP secretion in whole taste buds

ATP is believed to be an excitatory transmitter between taste buds and gustatory sensory afferent fibers [4], [19]. Studies from our lab and others have demonstrated that serotonin, released from Presynaptic (Type III) cells during taste stimulation, reduces ATP secretion. This serotonergic inhibition is generated by 5-HT_1A_ receptors on Receptor (Type II) cells [3], [28], [35]. Because glutamate triggers 5-HT secretion from taste buds (Fig. 2), and because 5-HT inhibits Receptor cells, we tested whether the net effect of glutamate stimulation might be to depress ATP secretion during taste stimulation. If so, this would suggest that the ultimate function of glutamate would be to decrease gustatory responses and transmitter (ATP) secretion in taste buds.

**Figure 3:**
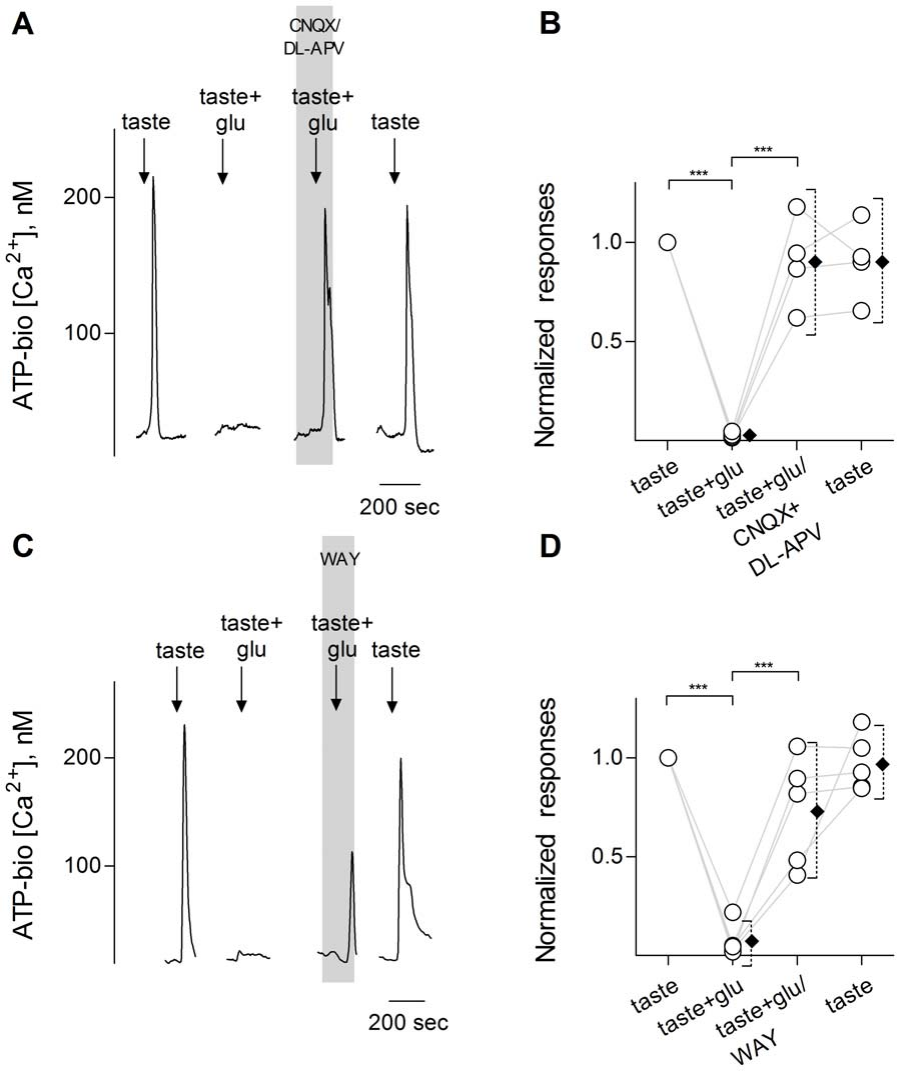
Serotonin, released during glutamate stimulation, inhibits taste buds. ATP biosensors were used to monitor taste-evoked transmitter release from taste buds. **A**, Traces show responses from a biosensor positioned near an isolated taste bud to measure ATP release elicited by taste stimulation. A sweet-bitter taste mix (↓, taste; 1 mM sucralose, 0.1 mM SC45647, 10 µM cycloheximide, 1 mM denatonium) evoked ATP release (biosensor response) that was inhibited by 100 µM glutamate (↓, taste+glu). Glutamate-evoked inhibition of ATP secretion was fully restored by adding combination of CNQX (30 nM) and DL-APV (15 µM) (present throughout the shaded area) to the bath. **B**, Summary of data. Open circles show normalized peak biosensor responses triggered by taste, taste+glutamate, taste+glutamate in the presence of CNQX and DL-APV, and finally, a repeat taste stimulus. As in Fig. 2, offset filled symbols show mean ± 95% CI, ***, p<0.001, repeated measures ANOVA, N = 4). **C**, In another experiment, a sweet-bitter taste mix (↓, taste) evoked ATP release (biosensor response) that was inhibited by 100 µM glutamate (↓, taste+glu). Glutamate-evoked inhibition of ATP secretion was partially reversed by adding WAY100635 (WAY, 10 nM, present throughout the shaded area), a 5-HT_1A_ antagonist, to the bath. **D**, Summary of data. Open circles show normalized peak biosensor responses of each experiment triggered by taste, taste+glutamate, and finally taste+glutamate in the presence of WAY100635. Offset closed symbols show mean ± 95% CI. ***, p<0.001, repeated measures ANOVA, N = 5).

Glutamate (100 µM) significantly and reversibly reduced ATP secretion evoked by a sweet/bitter taste mix (Fig. 3). Importantly, a combination of CNQX and DL-APV nearly fully recovered the inhibition of glutamate-induced reduction of ATP secretion evoked by taste stimuli (Figs. 3A, B). These results suggest that glutamate (100 µM) mainly activates ionotropic, not metabotropic glutamate receptors. To confirm that the inhibitory actions of glutamate were indirect and mediated, at least in part, by 5-HT, we blocked 5-HT_1A_ receptors with 10 nM WAY100635, a selective antagonist. WAY100635 significantly reversed glutamate-mediated inhibition of taste-evoked ATP secretion (Fig. 3C, D). This finding is consistent with the interpretation that glutamatergic inhibition is mediated largely by serotonin. The observation that WAY100635 did not entirely rescue taste-evoked ATP secretion in the presence of glutamate (Fig. 3C, D) has two explanations. First, to enable more rapid recovery after washout, we applied a low concentration of WAY100635 (10 nM). Second, and more importantly, we recently showed that Presynaptic cells also secrete another inhibitory paracrine transmitter, GABA [8]. WAY100635 only blocks the serotonergic contribution to taste-evoked inhibition of ATP release. We did not explore glutamatergic stimulation of GABA from Presynaptic cells in the present study.

## Discussion

This report shows that glutamate, kainic acid, and NMDA stimulate glutamatergic synaptic receptors and evoke Ca^2+^ responses in Presynaptic (Type III) taste cells. Most importantly, however, these agonists also stimulate taste buds to secrete 5-HT, a Presynaptic cell transmitter which inhibits Receptor cells by paracrine activation of 5-HT_1A_ receptors [3], [28], [35]. Glutamate-evoked release of 5-HT in the taste bud inhibits the response of taste buds to gustatory stimulation, and in particular reduces taste-evoked ATP secretion. Based on the presence of vesicular glutamate transporters in nerve fibers innervating taste buds, the findings support the notion that glutamate is an efferent transmitter for the peripheral end organs of taste [25]. Our study demonstrates that the net effect of efferent glutamatergic input is to initiate serotonergic inhibition within taste buds and to reduce taste-evoked release of the excitatory taste transmitter, ATP.

Serotonin, and more recently, GABA, have been shown to be inhibitory paracrine transmitters that are released from Presynaptic (Type III) taste cells. They both strongly depress Receptor (Type II) cell responses to taste stimulation [8], [28], [36]. Presently, the specific anatomical sites and mechanisms whereby these two inhibitory transmitters are released from Presynaptic cells are not known. Presynaptic cells form ultrastructurally identifiable synaptic contacts with nerve fibers [37], [38] and these synapses may release serotonin [39]. However, it is not known whether these same synapses are responsible for the paracrine secretion of GABA and serotonin. Detailed mechanisms for, and sites of paracrine transmitter secretion in taste buds remain relatively unexplored topics. Further, little is known about efferent synapses in taste buds. Ultrastructural features believed to be associated with efferent synapses, such as subsynaptic cisternae, are present in taste cells [40], but their relevance to efferent transmission has been questioned [41]. Further, as discussed below, the release of glutamate in taste buds is likely to resemble secretion of glutamate from peripheral terminals of nociceptive free nerve endings, that is, without well-defined synaptic structures.

Our findings suggest that altering serotonin in taste buds should affect taste sensitivity. Accordingly, researchers have attempted to perturb peripheral serotonin levels in humans and experimental animals and record changes in taste thresholds. Specifically, taste thresholds for sucrose and quinine were significantly reduced in human subjects after administering paroxetine, a selective serotonin re-uptake inhibitor [42]. The taste effect was measured at a point when pharmacokinetic studies had shown a peak elevation of plasma serotonin. In contrast, no taste behavior changes were observed when rats were administered paroxetine [43]. However, plasma serotonin concentrations and access of the monoamine to taste buds in the rat were unknown factors in those experiments. Parenthetically, mutant mice lacking 5-HT_3_ receptors showed no obvious taste deficits [4]. However, serotonergic inhibition in taste buds is mediated by 5-HT_1A_ receptors [28]. The relevance of knocking out 5-HT_3_ receptors in taste buds is uncertain. In short, the role of serotonin as a paracrine transmitter in taste behavior remains somewhat controversial and the tests are incomplete.

An important concern when testing glutamate on taste buds is whether the amino acid is activating basolateral synaptic receptors or apical taste receptors, or both. Glutamate is a prototypic gustatory stimulus that elicits umami taste. However, the threshold for activating glutamate taste receptors (>1 mM; [31]–[34] exceeds that used in the present study (100 µM), reinforcing our confidence that the responses we observed are due to activation of basolateral synaptic receptors. This conclusion is entirely consistent with prior investigations of glutamatergic synaptic receptors in taste buds and based on investigations of intact taste buds in a lingual slice preparation [24]. Moreover, the cells that responded to glutamate in the present study, Presynaptic (Type III) cells, do not express the taste receptors for umami; Receptor (Type II) cells express these taste receptors [17], [33], [44].

The overall proportion of taste cells showing increased intracellular Ca^2+^ in response to glutamate in our study is comparable to what has previously been reported. Caicedo et al. [24] observed that overall, 26% of taste cells in rat lingual slices showed increased intracellular Ca^2+^ in response to 300 µM glutamate. This would be comparable to the 25% incidence we report here for isolated mouse taste cells. Caicedo et al. [24] also showed a larger proportion of KA-responsive taste cells as compared to NMDA- responsive taste cells. The specific taste cell types were not identified in that study. When limited to identified Presynaptic cells in mouse taste buds, we found ∼50% were glutamate-sensitive. Vandenbeuch et al. [25] observed that ∼55% of Type III (Presynaptic) cells responded to glutamate. However, Niki et al. [34] observed a significantly lower incidence of glutamate-responsive taste cells when the amino acid was applied basolaterally at synaptic concentrations and changes in spontaneous impulse firing rate were monitored. They reported that 100 µM glutamate increased the baseline firing rate in only ∼10% of mouse fungiform taste cells. Although those data may seem to run counter to the findings of Caicedo et al. [24], Vandenbeuch et al. [25], and the present results, the explanation is straightforward. Niki et al. [34] investigated fungiform taste buds from mice and used electrophysiological recordings of spontaneous impulses. Fungiform taste buds have a significantly lower population of serotonergic Presynaptic (Type III) cells [45], [46] than the circumvallate taste buds used in the other cited reports, including the present study, making it less likely that inhibition mediated by Presynaptic cells would be observed. Moreover, Vandenbeuch et al. [25] and we selectively identified and recorded from Presynaptic (Type III) taste cells. Niki et al. [34] recorded from all taste cells that generated impulses, regardless of taste cell type. Finally, Caicedo et al. [24], Vandenbeuch et al. [25] and the present report measured Ca^2+^ transients in taste cells; Niki et al. [34] recorded impulse activity and designed their experiments specifically to investigate the excitatory effects of glutamate. Given all these differences, it is unlikely that Niki et al. [34] would have observed the inhibitory glutamatergic taste bud responses reported here.

**Figure 4:**
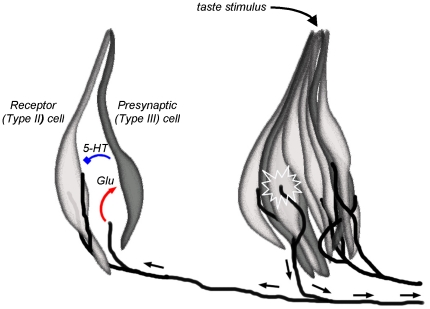
Diagram showing postulated mechanism for glutamate as an efferent transmitter in taste buds. Glutamate-stimulated release of serotonin might explain interpapillary inhibition that has been reported by others [50]–[54]. Two taste buds are depicted. Taste stimulation of the taste bud on the right activates a sensory afferent fiber that propagates signals centrally (small arrows to right at bottom) as well as laterally (interpapillary) to an adjacent taste bud via afferent branches(small arrows to left at bottom). Glutamate, released from an afferent axon branch (red arrow, left), activates NMDA and KA receptors on Presynaptic (Type III) taste cells. Glutamatergic stimulation of Presynaptic cells triggers these cells to secrete 5-HT, which inhibits ATP release from Receptor (Type II) cells (blue symbol) [28].

One interpretation of glutamatergic actions on taste buds is that glutamate is an efferent transmitter [25]. However, that interpretation need not necessary imply descending efferent control, but instead might involve local feedback from branches of sensory afferent fibers. That is, in addition to communicating signals from taste buds to the brain, sensory afferent fibers may also secrete glutamate as a peripheral feedback signal, similar to axon reflexes in peripheral nociceptive sensory afferent fibers [47]. Indeed, C fiber nociceptors are believed to secrete glutamate at their peripheral terminals during pain activation [48], [49]. In the lingual epithelium, branching sensory afferent fibers innervate 2 or more neighboring taste buds. These branching fibers appear to mediate inhibitory peripheral interpapillary interactions [50]–[54] but to date there have not been any convincing explanations for the mechanisms underlying this inhibition. The present report showing how a postulated efferent release of glutamate ultimately inhibits taste buds (i.e., via glutamate-evoked 5-HT release) may provide one answer. Figure 4 summarizes this scenario in a schematic diagram.
